# Predicting 3D soft tissue dynamics from 2D imaging using physics informed neural networks

**DOI:** 10.1038/s42003-023-04914-y

**Published:** 2023-05-18

**Authors:** Mohammadreza Movahhedi, Xin-Yang Liu, Biao Geng, Coen Elemans, Qian Xue, Jian-Xun Wang, Xudong Zheng

**Affiliations:** 1 Mechanical Engineering Department, University of Maine, Orono, ME 04469 USA; 2 Aerospace and Mechanical Engineering Department, University of Notre Dame, Notre Dame, IN 46556 USA; 3 Mechanical Engineering Department, Rochester Institute of Technology, Rochester, NY 14623 USA; 4 Department of Biology, University of Southern Denmark, Odense M, 5230 Denmark

**Keywords:** Biophysical methods, Diagnostic markers

## Abstract

Tissue dynamics play critical roles in many physiological functions and provide important metrics for clinical diagnosis. Capturing real-time high-resolution 3D images of tissue dynamics, however, remains a challenge. This study presents a hybrid physics-informed neural network algorithm that infers 3D flow-induced tissue dynamics and other physical quantities from sparse 2D images. The algorithm combines a recurrent neural network model of soft tissue with a differentiable fluid solver, leveraging prior knowledge in solid mechanics to project the governing equation on a discrete eigen space. The algorithm uses a Long-short-term memory-based recurrent encoder-decoder connected with a fully connected neural network to capture the temporal dependence of flow-structure-interaction. The effectiveness and merit of the proposed algorithm is demonstrated on synthetic data from a canine vocal fold model and experimental data from excised pigeon syringes. The results showed that the algorithm accurately reconstructs 3D vocal dynamics, aerodynamics, and acoustics from sparse 2D vibration profiles.

## Abstract (web summary)

A hybrid physics-informed neural network and differentiable learning algorithm integrates a recurrent neural network model of 3D continuum soft tissue with a differentiable fluid solver to infer 3D tissue dynamics from sparse 2D images.

## Introduction

Tissue dynamics in many organs play critical roles in physiological functions, such as the contraction of the atrial and ventricles in heart pumping, heart valve dynamics in blood circulation, and vocal-fold dynamics in voice production. The diagnosis and treatment of diseases often include an assessment of tissue dynamics. In recent years, medical imaging has undergone a major development toward capturing 3D tissue structure with much higher resolution and less noise and artifacts. However, real-time high-resolution imaging of 3D tissue dynamics remains a challenge due to factors including accessibility and temporal/ spatial resolution of measurement and image reconstruction.

Vocal-fold vibration during human phonation represents one of the greatest challenges in acquiring 3D dynamics. Upon phonation, the pair of vocal folds are adducted to close the glottis. As air is forced from the lungs, the adducted vocal folds are pushed apart by air pressure, and if the conditions between air pressure and tissue elastic force are right, the vocal folds are set into sustained vibrations. In clinics, the dynamics of the vibration is an important metrics for voice diagnosis and is commonly assessed using imaging techniques such as strobolaryngoscopy and high-speed endoscopy. However, endoscopy is limited in that it captures only a 2D top view of the vocal-fold motion without taking into account the vertical component, which has been shown by research to be important for phonation^1–5^. Several techniques have been explored toward measuring 3D dynamics of vocal-fold vibration. For examples, high-speed camera tracking small markers sutured on tissue surface was attempted in in vivo canine larynx^6^, high-frame-rate ultrasound was used to estimate body-layer movement of the vocal folds^7^, a laser system combined with high-speed cameras was explored for getting point-wise information of 3D motion^8^, high-speed stereo-endoscope was developed to reconstruct 3D motion from two views from different angles^2^, and optical coherence tomography (OCT) was used to obtain high-speed, cross-sectional laryngeal imaging to quantify mucosal wave in the vertical direction^9^. Despite these developments, compromises are often made on temporal and spatial resolutions because of technical limitations of the sensors and the illumination needed for high-speed recording in wide voice frequency ranges from 80 to 1100 Hz^10^. However, real-time, high-resolution measurement of 3D vocal-fold dynamics in in vivo larynges has not been achieved.

Physics-informed neural network (PINN), as a recently developed class of deep learning models, shows great promise in inversely reconstructing 3D field solutions from sparse/indirect measurements^11^. The central idea of PINN is to use physics to inform the network training by penalizing the violation of physical laws and constraints, thus enabling sparse-label learning, assimilation of indirect data, and improved sample efficiency and generalizability. Since its appearance, PINN has been successfully applied in various scientific applications, including but not limited to aerodynamics^12–15^, biomechanics^16,17^, chemical systems^18–20^, and heat transfer^21,22^.

PINN can be potentially applied to reconstruct 3D tissue dynamics from 2D imaging. However, traditional PINNs face tremendous challenges in dealing with3D flow-structure interactions (FSI), which are constantly involved in tissue dynamics. First, the physics laws in a continuous PINN are evaluated at individual collocation points, the amount of which can easily become huge for large-scale 3D problems with a high-dimensional parameter space, making scalable training infeasible. Past PINN studies have been mostly focused on 2D problems^12,13,21,22^ with a few attempts on steady problems in 3D^23,24^. Recently, some studies showed improved scalability in discrete PINN schemes that combine classic numerical techniques with deep learning^25–29^. Second, the bi-physics nature of FSI, especially the non-smoothness across FSI interface, is difficult to capture by a classic neural network with a multilayer perceptron (MLP) structure. This becomes even more challenging when soft tissue is involved, which typically experiences large deformations with complex spatiotemporal dynamics. Third, traditional PINNs usually do not include temporal dependence of data, so they are very difficult to converge for problems with complex temporal dynamics. The challenge is further exacerbated when the network predictions are forced into the extrapolation regime, which is not uncommon for many-query applications of trained network models in forward or inverse uncertainty quantification.

In addition to the above three challenges related to 3D FSI, another challenge exists when building correspondence between network prediction and imaging data. Traditional PINNs are usually built upon direct one-to-one correspondence between data and network prediction. However, medical imaging often generates 2D deformation contours through slicing or projection. As a result, there is no direct correspondence between network predictions and measured data, which complicates the construction of the loss function.

In this study, we designed a hybrid PINN-differentiable learning algorithm to reconstruct high-resolution 3D vocal-fold motion from 2D endoscopic imaging. The algorithm integrates a recurrent neural network of a 3D continuum model of soft tissue and a differentiable fluid solver to address the above challenges. The algorithm was first validated against simulation data of vocal-fold vibration in a canine larynx. The prediction accuracy was evaluated by comparing the 3D deformation fields and key aerodynamic and acoustic quantities, including the mean and maximum flow rate, intraglottal pressure, and sound pressure level (SPL) and acoustic power. The algorithm was then validated against experimental data of self-vibrating bird syringes. Because 3D kinematics of the vibrating mass in the experiments were not available, a cross-validation was conducted by comparing the resulted acoustic quantities, including the SPL and acoustic power. We would like to note that the algorithm offers another advantage to be able to infer many other quantities due to the inclusion of the physics laws, such as tissue stress, which are otherwise very difficult/impossible to measure in experiments or clinics. The validation on these quantities was performed on the synthetic dataset, but not on the experimental dataset as they are not available. We would also like to note that even although the algorithm is demonstrated in laryngeal/syringeal dynamics in this study, it is designed for general 3D FSI problems for broad tissue dynamics applications. The algorithm can advance disease diagnosis by going beyond 2D dynamic criterion and expanding physical quantity metrics.

## Results

The details of the network architecture and numerical schemes can be found in the Methods section. In brief, to address the inherent nonlinearities resulting from fluid-solid coupling, the PINN loss is constructed purely based on the residuals of the governing equations of solid mechanics. To compute the fluid loading term on the right-hand side of the governing equation, a fully differentiable numerical fluid solver is integrated into the neural networks as a unified differentiable program. The integration of a differentiable numerical fluid solver into the neural network allows us to efficiently compute the fluid loading term, enabling us to achieve end-to-end differentiability and optimize the model parameters effectively. To enhance the scalability and convergence of the neural networks, the algorithm leverages the prior knowledge in solid mechanics by projecting the governing equation onto the numerical eigenmode space to reduce the infinite dimensions of the continuous solution space to a finite dimension of discrete search space. The dimensions of the problem are further reduced by only using truncated eigenmodes, which can effectively represent the whole dynamics with negligible errors. To better capture the temporal dependence of the FSI dynamics and enhance the predictive accuracy, a Long Short-Term Memory (LSTM)-based recurrent encoder-decoder connected with a FCNN is designed to learn the time history of modal coefficients, which, combining with eigenmodes, enable the spatiotemporal predictions of tissue dynamics.

### Synthetic data test

**Fig. 1:**
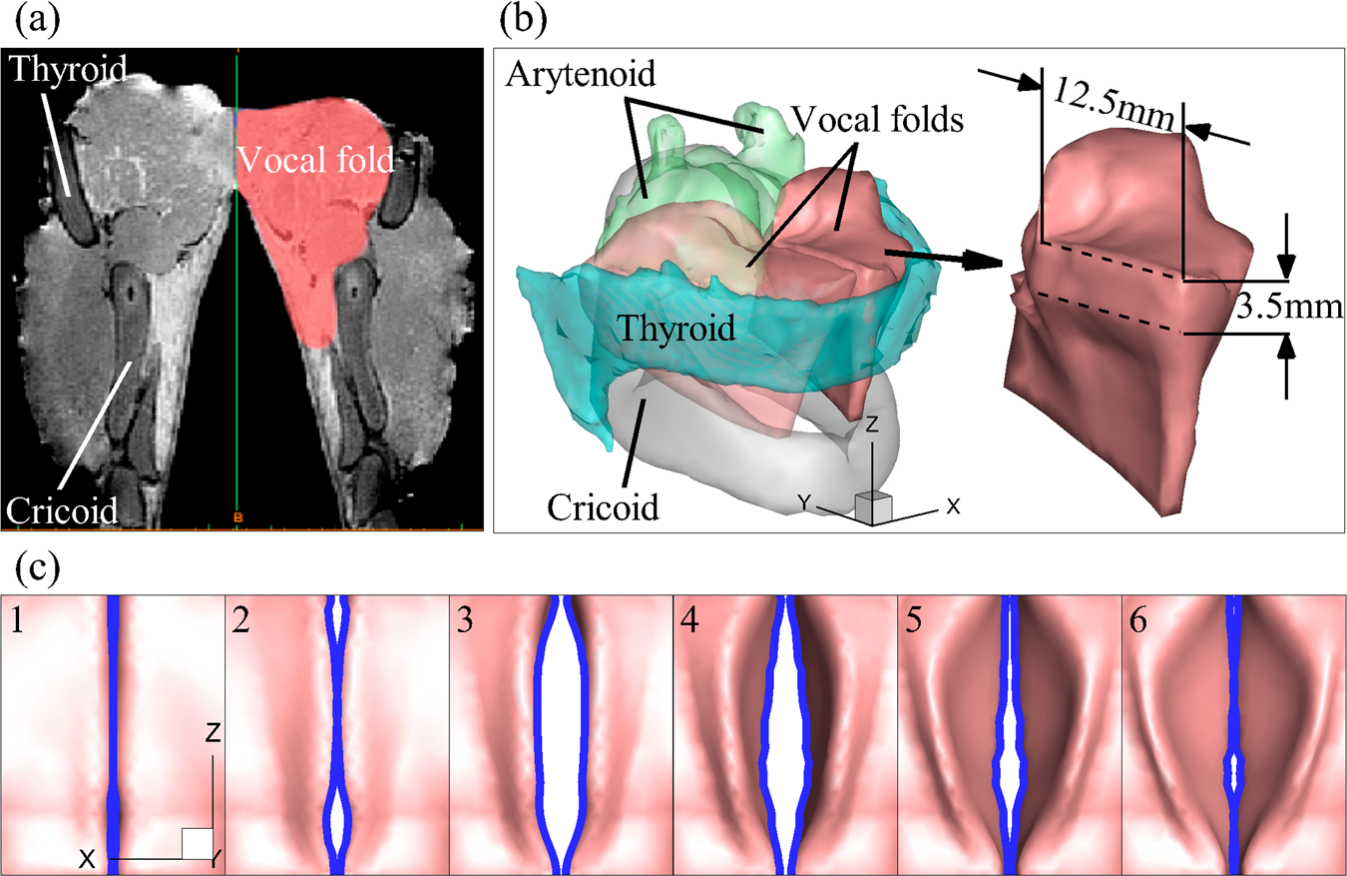
A canine larynx model reconstructed from MRI scans and the representative 2D glottal shapes used for training the PINN. **a** An MRI scan of the canine larynx in the mid-coronal plane, where different parts are annotated. **b** The reconstructed 3D model of the larynx. The vocal folds and cartilages are shown. One vocal fold with key dimensions is shown to the right. **c** The simulation results of vocal fold dynamics from the top view at six representative time instants during one vibration cycle when the glottis is open. The 2D glottal shapes are denoted by the blue lines. A total of 20 time-labeled 2D glottal shapes were extracted from one vibration cycle and used as the input data to the PINN.

We first tested the algorithm using synthetic data which is high-fidelity simulations of 3D vocal-fold dynamics in a canine larynx. The advantage of synthetic data test is that the ground-truth values of quantities of interest are available for an accurate comparison. Figure 1b shows a 3D canine laryngeal model with components of realistic geometries reconstructed from high-resolution MRI scans, one image of which is shown in Fig. 1a. The model contains all the major cartilages, intrinsic muscles, and vocal-fold tissues. The vocal fold with key dimensions is shown to the right of Fig. 1b. FSI simulations of vocal fold dynamics in this model were reported in a previous study, and the simulations reproduced the key features of glottal aerodynamics and vocal fold dynamics observed in experiments. The details of the simulations are referred to ref. ^30^.

Figure 1c shows the simulation results of vocal fold dynamics at six representative time instants during one vibration cycle when the glottis is open. The figures are from the top view, which is consistent with the endoscope/laryngoscope view in clinics. The blue lines denote the projected 2D glottal shapes from the top view, which are used as the input data to the PINN. Practically, such input data can be obtained by segmenting the vocal fold edges visible in endoscopic images, a task for which various automated algorithms have been proposed^31–33^. The vibration frequency of this model is 143 Hz. We extracted 20 time-labeled 2D glottal shapes over one vibration cycle for the training of the PINNs, which resulted in a sampling rate of 2.5 kHz, on par with the 1–4 kHz sampling rates of typical laryngeal endoscopes in the clinical setting, e.g., flexible fiberoptic endoscope or high-speed rigid endoscope. These 2D shapes were fed to the PINN for computing the data loss during training. Additionally, a numerical computation of the eigenmodes of the vocal fold model was conducted and the lowest 100 eigenmodes along with their eigenfrequencies were fed to the PINN for computing the equation loss during training.

**Fig. 2:**
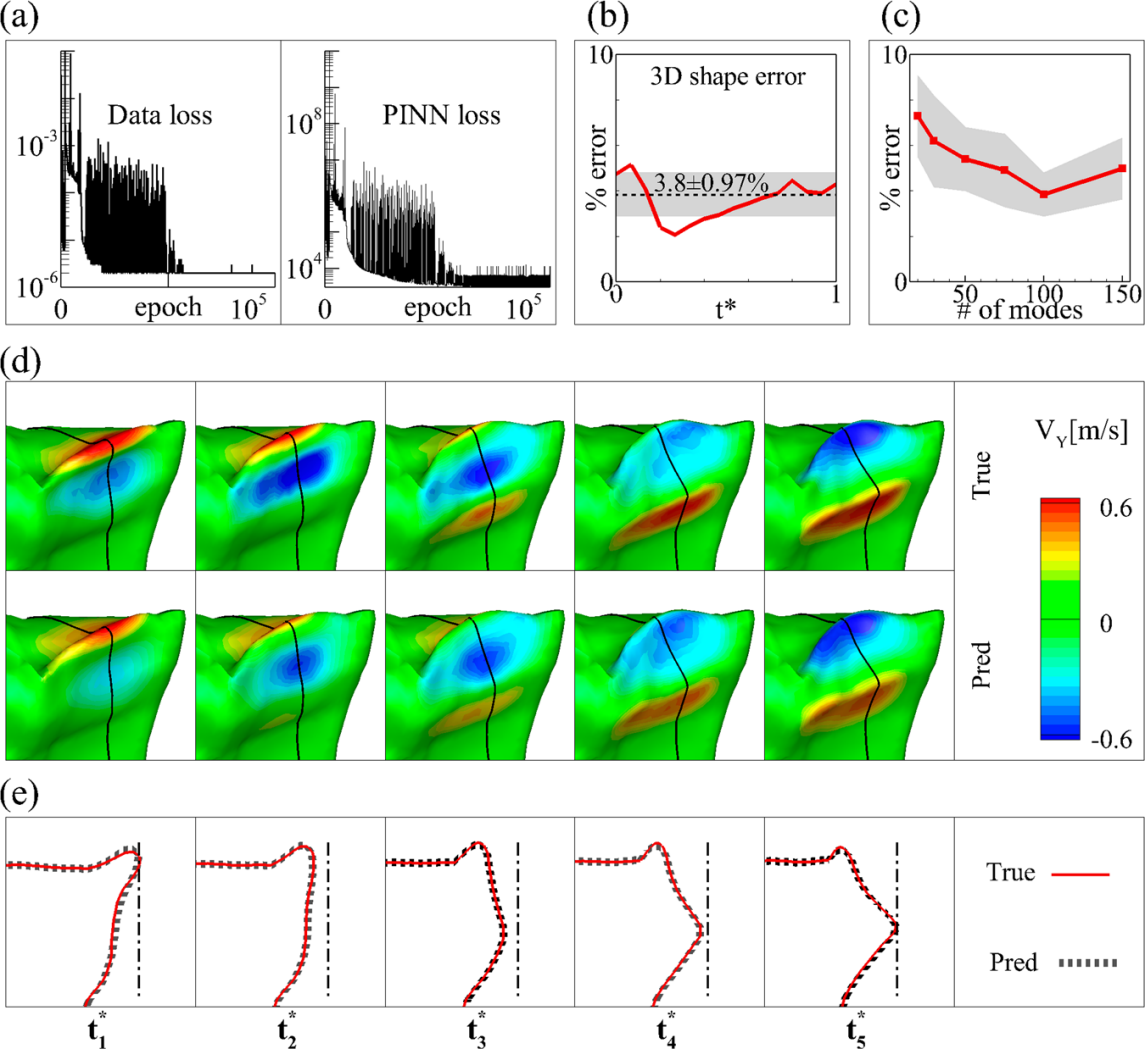
PINN training results of 3D vocal fold dynamics in the canine larynx. **a** The history of data (left) loss and equation (right) loss during the training. **b** The prediction error of 3D vocal fold shapes over one cycle. The dashed line and the shaded area indicate mean and standard deviation (SD) over a cycle, respectively. **c** The mean and SD of the prediction error of 3D vocal fold shapes versus the number of eigenmodes adopted. **d** Comparison of 3D vocal fold shapes and vertical velocity component contour between the PINN prediction and ground truth at 5 representative time instants in a cycle (shown in Fig. 3a). **e** Comparison of the vertical profile of the vocal fold at the mid-coronal plane between the PINN prediction and ground truth at the corresponding time instants.

Figure 2 shows the PINN prediction results of 3D vocal fold dynamics. Figure 2a shows that both the data and equation losses of the PINN training converged after around ${6\times 10}^{4}$ epoch. The data loss is calculated based on the mean squared error between the true and predicted 2D profiles. The PINN loss is calculated based on the mean squared error between the left-hand side (LHS) and right-hand side (RHS) of the modal dynamic equation for each eigenmode. Figure 2b shows the prediction error of the 3D vocal fold shapes over one vibration cycle, represented by the L2 norm of the difference of the displacement vectors between the PINN prediction and FSI simulation normalized by the norm of the displacement vector from the FSI simulation. The error is between 2.0 and 5.1% over one vibration cycle with the mean value of 3.8% and standard deviation (SD) of 0.97%. We also examined the sensitivity of the prediction to the number of eigenmodes adopted in the network by creating five more cases with 20, 30, 50, 75, and 150 eigenmodes. Figure 2c shows that the error decreases quickly with the increase of the number of the modes. The error is highest at 7.3% with 20 modes and converges to 3.8% with 100 modes.

Figure 2d compares the 3D vocal fold shapes and vertical velocity component contour, and Fig. 2e compares the vertical profile of the vocal fold at the mid-coronal plane between the PINN prediction and FSI simulation (ground truth). These figures show that the 3D vibratory dynamics and vertical velocity contours are accurately predicted by the PINN. The maximum amplitudes of the lateral and vertical motion on the medial surface (see a summary in ref. ^34^) are 2.57 mm and 1.75 mm in the ground truth, and 2.53 mm and 1.68 mm predicted by PINN. The prediction errors and SD are −1.6 ± 2.9% and −3.6 ± 3.7%, respectively. The maximum vertical velocity is 1.03 m/s in the ground truth and 0.95 m/s predicted by PINN. The prediction error and SD is −7.7 ± 7.6%. Note that the standard deviation is calculated using multiple measurements around the mid-coronal region of the medial surface.

Due to the inclusion of the physics laws in the network, PINN also allows inferring other physical quantities that are not available in the training data. We demonstrate this ability by comparing several important aerodynamics and acoustics quantities predicted by our PINN algorithm to those available from the FSI simulations.

**Fig. 3:**
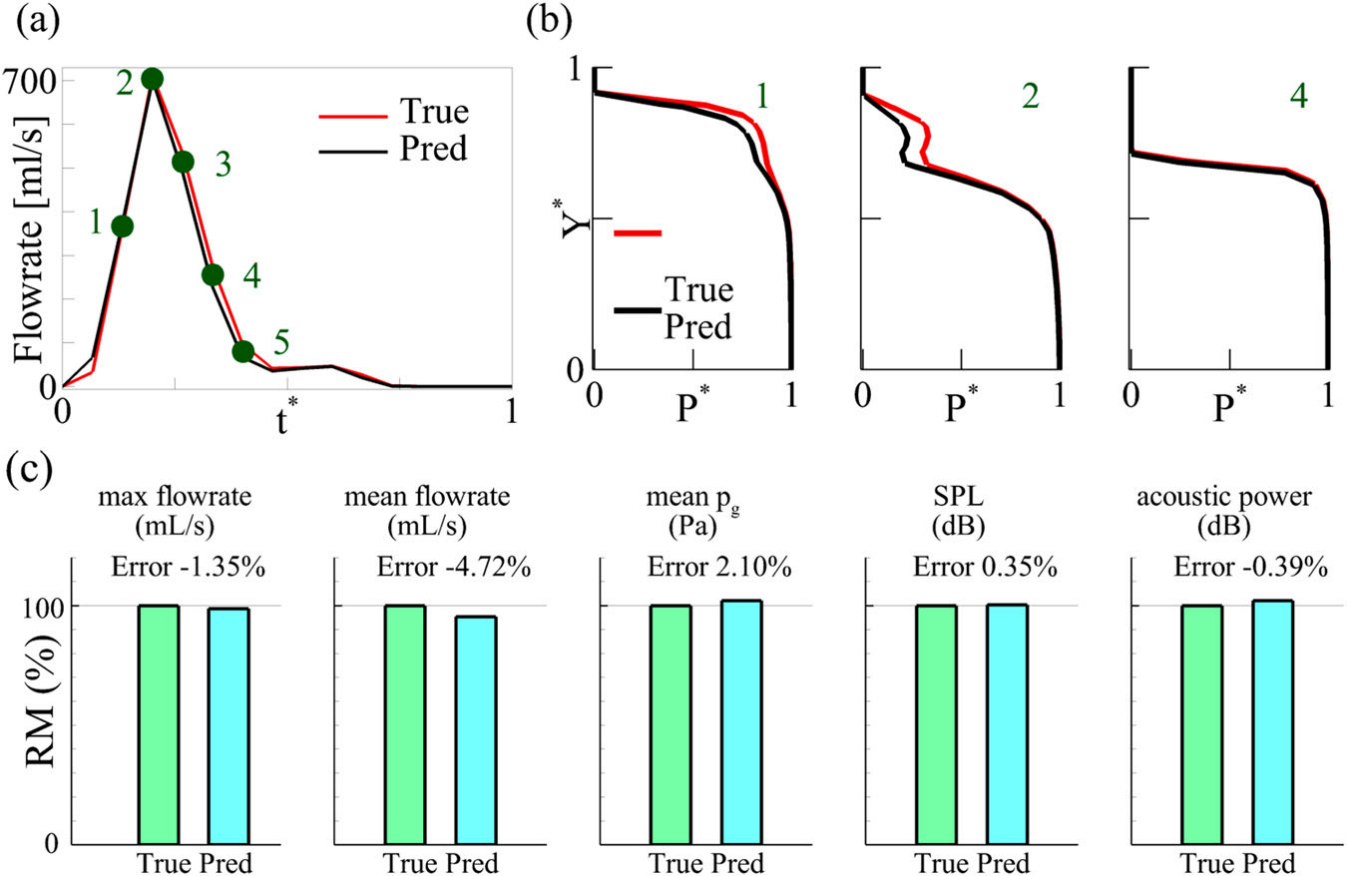
Comparison of aerodynamics and acoustics quantities between the PINN prediction and FSI simulation (ground truth) in the canine larynx. **a** Glottal flow rate waveform over one vibration cycle. **b** Intraglottal pressure at three time instants within a cycle. **c** Relative amplitude (RM) of key quantities with errors.

Figure 3a compares the temporal history of glottal flow rate over one vibration cycle and Fig. 3b compares the intraglottal pressure distribution along the streamline at three representative time instants between the ground truth and PINN predictions. The three time instants are denoted in Fig. 3a, representing glottis opening, maximum opening and closing, respectively. The PINN accurately predicted the opening and closing quotients and the peak flow rate. The time-mean error of the flow rate is 1.7% with 2.4% standard deviation. The distribution of the intraglottal pressure along the streamline is also accurately predicted as denoted in Fig. 3b. The time-mean error of the mean intraglottal pressure is 2.1% with 1.6% standard deviation. Figure 3c compares several important aerodynamics and acoustics quantities, including the peak flow rate, mean flow rate, mean intraglottal pressure, SPL, and acoustic power. The prediction errors (and ±SD when applicable) are −1.35%, −4.72%, 2.10 ± 1.6%, 0.35%, and −0.39%, respectively, highlighting the comprehensive predictive capability of the current algorithm.

### Experimental data

**Fig. 4:**
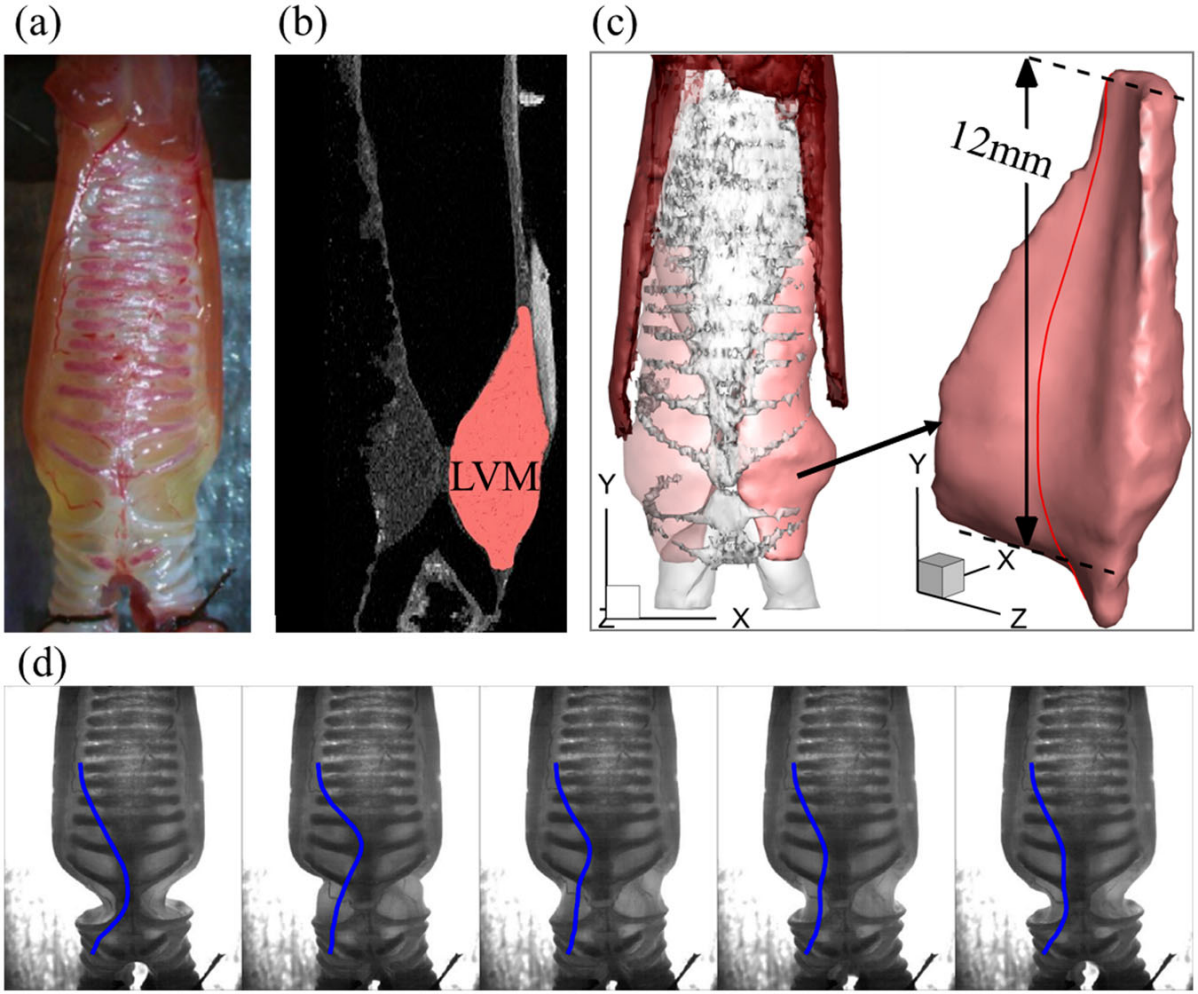
A syrinx model reconstructed from DiceCT scans and the representative 2D lateral vibratory mass (LVM) shapes for training the PINN. The same type of data and models were generated for other three pigeon syringes. **a** The photo of the excised syrinx. **b** A DiceCT scan of the syrinx. One LVM was annotated. **c** The 3D computational model reconstructed from the DiceCT scans including the pair of LVMs and the surrounding cartilages. A detailed view of the left LVM is shown to the right. The red line shows the mid-coronal cross-section of the LVM. **d** Representative snapshots of LVMs vibrations during one vibration cycle extracted from high-speed videos. The blue lines denote the 2D profiles of the LVMs which were manually annotated.

We further tested the algorithm on the in vitro experimental data of pigeon syringeal dynamics and sound production. The bird syrinx was shown to exhibit a similar dynamics as the human vocal fold, represented by a mucosal wave propagation on the tissue surface. The bird syrinx also bears the same underlying physics of voice production as the human larynx^35^. Validating the algorithm on bird syrinx dynamics provides confidence on the applicability of the algorithm on human larynx dynamics. We obtained high-speed images of lateral vibratory mass (LVM, the vocal fold analog in syrinx) vibrations in four pigeon syringes along with simultaneous acoustics measurements. Figure 4a shows the photo of the excised syrinx of one pigeon. Figure 4b is the DiceCT scan of the syrinx with the LVM annotated. Figure 4c shows the 3D computational model of the syrinx reconstructed from the DiceCT scans. The model includes a pair of LVMs and the cartilages surrounding them. Figure 4d shows representative snapshots of LVMs vibrations during one vibration cycle extracted from high-speed videos. Contrary to the laryngoscope view in the human larynx, the images were obtained from the ventral-dorsal view. The blue lines denote the 2D profiles of the LVMs, which were manually annotated. These 2D profiles were the input data for PINN training. The same approach was applied to the other three pigeons. Detailed information on the experiment, syringe models, and training parameters can be found in “Methods”.

**Fig. 5:**
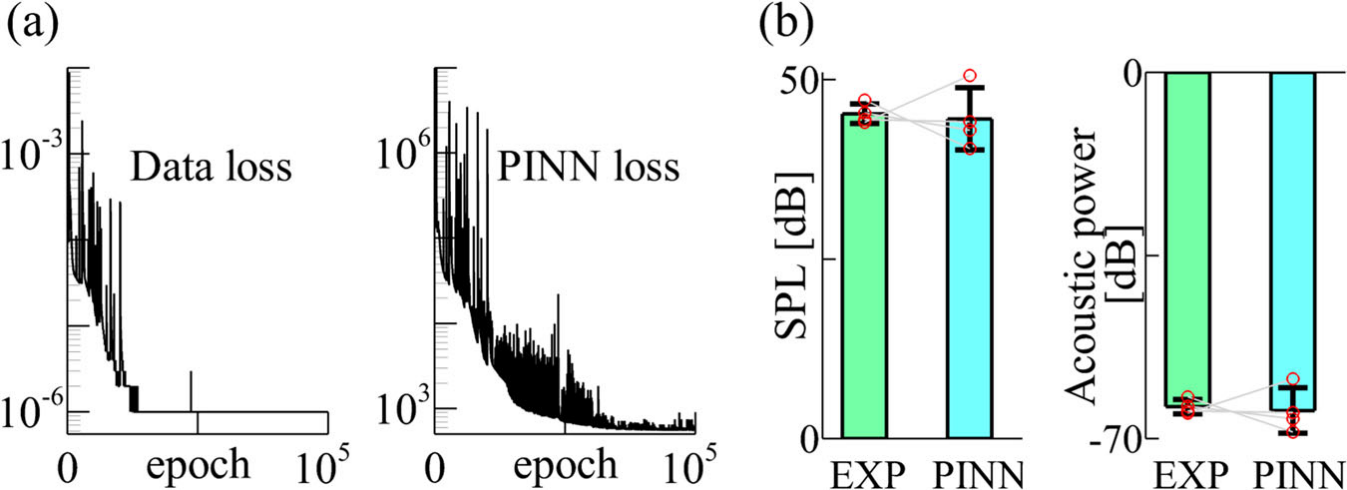
PINN convergence histories and cross-validations against acoustic measurements from excised syrinx experiments. **a** The history of data (left) and equation (right) loss during PINN training for one of the syringeal models. Data loss and PINN loss converged after around 27,000 and 70,000 epochs, respectively. **b** Comparison of acoustic parameters between PINN prediction and experimental data. The bar shows the mean value, and the error bar represents the standard deviation for four subjects. The gray straight lines indicate the correspondence of each subject between experimental measurement and PINN prediction. Individual data points are marked as red circles.

The PINN was trained on a single NVIDIA A100 GPU for about 7 h and successfully converged for each syrinx model. Figure 5a shows the convergence of the data and equation losses of the network for one syrinx as an example. Different from the synthetic data, the full 3D LVMs dynamics were not available from the experiment, so a direct validation on 3D dynamics is not possible. We thereby cross-validate the algorithm by comparing the acoustic quantities between PINN prediction and experiment. Figure 5b shows the comparison of the SPL and acoustic power. In each subfigure, the solid columns represent the mean values over the four syringes, and the bars represent the standard deviations. Good agreement between the PINN prediction and experimental measurement is observed. The difference in the mean values is 1.6% and 1.1% for SPL and acoustic power, respectively. The SD for SPL and acoustic power are both ±1.4 dB in the experiment, and both ±4.3 dB in PINN prediction. As the sound production is mainly modulated by the syringeal dynamics, it implies that the syringeal dynamics has been correctly predicted.

**Fig. 6:**
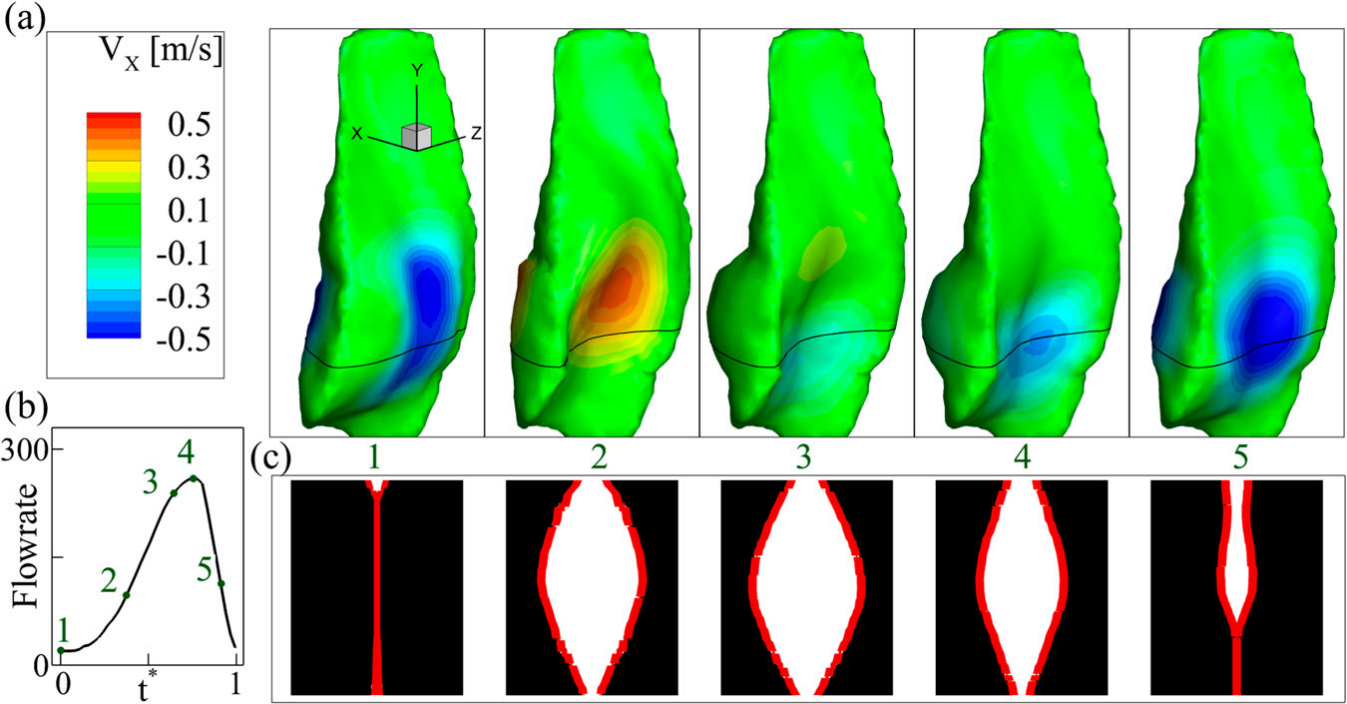
PINN predicted 3D LVM dynamics and flow rate from the syringeal model shown in Fig. 4. **a** 3D LVM shapes and lateral velocity contour at five representative time instants over one vibration cycle. The five time instants are denoted in (**b**) which is the flow rate over one cycle. **c** Syringeal opening area at a horizontal section (indicated by the black lines in (**a**) at the same five time instants. The red lines depict the boundary of the LVMs.

Figure 6 shows the 3D LVM shape and flow rate predicted by the PINN. These results demonstrate that the actual vibration of the LVMs is highly 3D and the PINN provides an innovative way to reproduce the 3D shapes and other physical data which otherwise are unavailable experimentally. Figure 6a depicts the 3D LVM shapes at five time instants over one vibration cycle. The color contour represents the lateral velocity with negative values (blue) indicating medial-ward motion and positive values indicating lateral-ward motion. Figure 6b shows the predicted syringeal flow rate with the numbers denoting the five corresponding time instants in (a). Figure 6c shows the profile of the two LVMs at a horizontal plane (indicated by the black lines in (a)) at the same time instants, illustrating the motion in the longitudinal (third) direction. Through the cycle, a strong wave propagation in the inferior-superior (caudal-cranial) direction with the inferior aspect leading the motion can be observed. Meanwhile, a strong motion in the longitudinal direction is observed. The LVM generally assumes a half-wavelength mode in the longitudinal direction while some phase differences can be observed during the closing phase (instant 5).

## Discussion

We presented a hybrid PINN-differentiable learning algorithm that integrates a recurrent neural network model of 3D continuum soft tissue with a differentiable fluid solver to infer the 3D flow-induced tissue dynamics and other physical quantities from sparse 2D profile measurements. The algorithm addresses the inherent challenges of PINN for inferring 3D FSI problems, including the convergence difficulties associated with high computational cost, the nonlinearity of FSI coupling, and lacking direct correspondence between measurement and network outputs. With this hybrid PINN-differentiable learning framework, we can reconstruct 3D FSI dynamics by matching the 2D image data, which is an inverse data assimilation problem per se. In this context, traditional numerical solvers fail to work alone, unless they are combined with an inversion algorithm, e.g., adjoint method, Bayesian sampling, or genetic algorithms, which are either heavily code-intrusive or require massive forward numerical simulations. Although the hybrid PINN model and traditional forward solvers are not directly comparable in scenarios of data assimilation and inverse modeling, the computational costs for training and evaluation of the hybrid PINN model are provided, which are 7 h and less than one second on a single GPU card, respectively. Compared to forward FSI simulation using traditional solver, which takes 2 CPU-hours, the proposed hybrid PINN has significant computational advantages when large number of model queries are required. The accuracy of the prediction was tested using synthetic and experimental datasets of canine laryngeal and bird syringeal dynamics. In the validation against the synthetic simulation data of canine laryngeal dynamics, we showed that the prediction errors and SD of 3D displacement fields are 3.8 ± 0.97% and that key aerodynamics and aeroacoustics quantities are all within 5% (Fig. 3c) with small deviation showing that the errors are not statistically significant. In the validation against the experiment data of bird syringeal dynamics, due to the lack of 3D dynamics data, we did the cross-validation by comparing the acoustic predictions. We showed that the prediction errors of acoustics quantities are within 2%. Standard deviation of the errors among multiple pigeon subjects are also small (Fig. 5b).

In the current study, we demonstrated the algorithm for its application in predicting laryngeal/syringeal dynamics; however, it is designed to be transformative for broad applications involving 3D FSI, such as cardiovascular dynamics, heart valve dynamics, animal flight/swimming dynamics, etc. While more rigorous validations against experimental and clinical datasets are needed in future, the algorithm has potential to impact the medical field by advancing disease diagnosis beyond the current 2D dynamics criterion. As the algorithm matures, it has the potential to expand the range of measurable quantities in both experimental/clinical research, therefore enhancing the research capabilities.

Another important potential impact of the algorithm to the medical field is that it allows inferring many physical quantities that are otherwise very difficult/impossible to obtain in clinics, such as glottal flow rate and vocal fold stresses in laryngeal dynamics examination. Currently, the glottal flow rate is estimated indirectly from pressure measurement at the mouth by using Rothenberg mask^36^, which cannot be used when endoscopy is performed, and no techniques are available for measuring vocal fold stresses. Previous data-driven methods were developed for automatic reconstruction of vibratory parameters from endoscopy images;^37^ however, the methods cannot predict other physical quantities. A few attempts were made toward predicting unmeasurable physical quantities using computer model integrating with data assimilation, yet the works have been limited to highly simplified vocal fold representations, e.g., lumped element model^38^ or 2D FEA representation^39^. The goal of this algorithm is to predict full 3D physical fields in anatomically realistic subject-specific models by integrating limited measurement data. We would like to note that once the algorithm is validated, the network needs to be trained on each subject’s data to obtain subject-specific coefficients in the model, which can then be used to reconstruct full physical fields, including the 3D vibration, aerodynamics, and acoustics, and predict at other conditions for the subject.

One critical aspect of the proposed algorithm is to project the governing equations onto the reduced eigenspace to effectively reduce the dimension of search space, facilitating network training and convergence. We chose to use modal dynamic analysis with reduced number of eigenmodes instead of a DNN autoencoder (AE) for dimension reduction for several reasons. First, our study deals with irregular 3D geometries represented by unstructured mesh data, making it infeasible to use a CNN-based AE as in ref. ^40^. Therefore, we need to apply graph neural network (GNN)-based AE, which is still in the early stage of development. Second, eigenspace methods have better generalizability compared to DNN-based AE, particularly when training geometry data is limited. Third, using a complex AE network could make the training even more challenging by introducing additional complexity to the overall structure.

Another critical aspect of our algorithm is to use a recurrent encoding-decoding discrete PINN architecture to resolve the convergence difficulty of stiff ODEs. After the projection to the reduced eigenspace, the original PDEs were transformed into a group of decoupled ODEs. Interestingly, the original continuous PINNs-based FCNN formulations are even more difficult to converge for stiff ODEs than some PDEs, which has been reported previously in other studies^41–43^. We have comprehensively studied the original continuous PINNs for given problems and also experienced convergence difficulties regardless of neural network structures. The difficulty may result from the stiffness of ODEs and nonlinearity in time, which lead to unbalanced back-propagated gradients between the loss of data and the loss of equation residual during training. We found that this difficulty of convergence on ODEs can be resolved by utilizing a recurrent encoding-decoding discrete PINN architecture, suggesting that an explicit representation of temporal dependence is essential for modeling the nonlinear dynamical systems governed by the ODEs. Another important aspect is the seamless integration of physics-based numerical solvers with deep neural networks within a differentiable programming framework, which allows gradient back-propagation throughout the entire program, enabling hard-encoded physics, training with partial/indirect data, and improved learning performance.

Finally, we would like to point out the limitations of our algorithm and suggest future works. First, the current algorithm relies on the material properties of the vocal fold a priori for computing the eigenmodes. In clinical settings, the in vivo material properties are typically unknown, which limits the current algorithm only to ex vivo studies. Yet, the algorithm can be expanded to infer the material properties by including the eigenmode computation in the training process. We are planning to implement this capability in future study. Second, a one-dimensional flow model is employed for simulating glottal aerodynamics. While it is a widely-adopted flow assumption in phonation models^44–46^, it poses constraints on its direct application on other applications, e.g., heart valve problems, where the three-dimensional vortex structures are inherently important. This limitation can be solved by implementing a more comprehensive/accurate flow solver with differentiable programming, where the fluid dynamics is described by the Navier–Stokes equation with trainable parameters and components, which can be learned from patient-specific data. Third, while the current algorithm only takes the 2D endoscopic/lateral images as inputs, it can be expanded to take synchronized multimodal inputs, such as acoustic measurements, by integrating additional physics laws and acoustic measurements. This will help to achieve faster convergence, higher accuracy, and the ability to be trained with even fewer 2D profiles as input. Fourth, a recurrent neural network-based structure is designed to capture complex temporal dynamics. While our hybrid neural solver effectively learns the temporal coherence of the input 2D images and the 3D structure, applying other state-of-the-art Seq2Seq network structures such as the Transformer^47,48^ may further improve the performance. Furthermore, some previous works have demonstrated the potential of applying PINN to denoise the measurements^49^, which can be helpful when the input 2D images are of low quality and require noise reduction. Lastly, the validation on the 3D dynamics in the current study is limited on the simulation data from a canine larynx, which cannot be translated to real-word vibrational behaviors of human larynx. Future studies are needed for more rigorous validations against experimental or clinical data from human larynx, such as high-speed video recording 3D vocal fold vibration in hemi-larynx^50^.

## Methods

The proposed algorithm tightly integrates a recurrent neural network model of 3D continuum soft tissue with a physics-based fluid solver within a differentiable programming framework to infer the 3D flow-induced tissue dynamics from its 2D projection measurements.

### Solid dynamics model

The equation of motion of a general system with damping under external forces can be presented as follows:^51^

1 $$\left[M\right]\left\{\ddot{U}\left(t\right)\right\}+\left[C\right]\left\{\dot{U}\left(t\right)\right\}+\left[K\right]\left\{U\left(t\right)\right\}=\left\{F\left(t\right)\right\}.$$

where $\left[M\right]$, $\left[C\right]$, and $\left[K\right]$ are mass, damping, and stiffness matrices of the system, respectively, and $F$ is the external force. Using the Rayleigh damping, $\left[C\right]$ can defined as a linear combination of $\left[M\right]$ and $\left[K\right]$:

2 $$\left[C\right]=\alpha \left[M\right]+\beta \left[K\right].$$

where $\alpha$ and $\beta$ are Rayleigh damping parameters. {$U$}, solution of Eq. (1), can be represented in a compact form using eigen decomposition,

3 $$\left\{U\left(t\right)\right\}=\mathop{\sum }\limits_{j}{b}_{j}\left(t\right)\left\{{U}_{j}\right\}.$$

where $\{{U}_{j}\}$ are eignmodes and ${b}_{j}\left(t\right)$ are modal coefficients. While continuous systems have infinitely many eigenmodes, only the lowest ones, usually the first 10 to 100 modes, are crucial in practice^51^. Considering that the intrinsic system dimension is usually low, the eigenmode series can be truncated to a finite number to approximate the solution $\{U\left(t\right)\}$. By choosing a suitable number of eigenmodes, this truncation error can be negligible. Eigenmodes and eigenfrequencies can be numerically calculated using packages such as ARPACK^52^, which is adopted in this study using the shift-invert mode. By substituting Eqs. (2) and (3) into Eq. (1), and multiplying the derived equation by the transpose of eigenmodes, and due to the orthogonality condition, Eq. (1) can be written as:

4 $${\ddot{b}}_{j}\left(t\right)+\left(\alpha +\beta {{\omega }_{j}}^{2}\right){\dot{b}}_{j}\left(t\right)+{{\omega }_{j}}^{2}{b}_{j}\left(t\right)={\{{U}_{j}\}}^{T}\left\{F\left(t\right)\right\}.$$

In Eq. (4), which is the central equation for the modal dynamics of the system, only coefficients ${b}_{j}\left(t\right)$ and $\{F(t)\}$ are functions of time. For a system with known properties, $\alpha$, $\beta$, ${\omega }_{j}$, and $\{{U}_{j}\}$, the equation can be solved to find ${b}_{j}\left(t\right)$ for each mode $j$, and finally $U\left(t\right)$ can be approximated by Eq. (3).

### Differentiable flow solver

The right-hand side fluid loading $\{F(t)\}$ is computed using a numerical fluid solver which is fully differentiable. For the phonation problem, it has been shown that the glottal flow dynamics can be reasonably represented by the modified (1D) Bernoulli equation^53–55^. In this model, fluid pressure $P\left(y\right)$ is a function of glottal channel area $A(y)$ as depicted in Eq. (5):

5 $$P\left(y\right)={P}_{{sub}}-\frac{1}{2}{\rho }_{{air}}{\left(\frac{Q}{A\left(y\right)}\right)}^{2}.$$

where $P\left(y\right)$ is the intraglottal pressure at the vertical location of $y$, $A(y)$ is the cross-sectional area, ${P}_{{sub}}$ is the subglottal pressure, $Q$ is the flow rate, and ${\rho }_{{air}}$ is the air density. The model assumes that flow separation occurs at the minimum glottal area and the flow pressure equals zero gage pressure downstream of the flow separation. Based on this assumption, the flow rate was calculated as:

6 $$Q=\sqrt{\frac{2{P}_{{sub}}}{{\rho }_{{air}}}}{A}_{\min }.$$

where ${A}_{\min }$ is the minimum glottal area. The implementation of the fluid solver is purely in PyTorch^56^, which supports automatic differentiation, enabling automatic computation of gradient for any computational graph. By leveraging the auto-differentiation backend of Pytorch, the differentiable fluid solver can be seamlessly coupled with neural networks, which can be trained as a unified program. This paradigm is also known as differentiable programming. The strength of differentiable programming is that the gradient for trainable parameters could pass through the numerical solvers to neural networks, which enables a sequence-to-sequence (Seq2Seq) training. When compared to next-step models, Seq2Seq training could significantly improve the error accumulation issue and leads to a much more stable long-term prediction. Therefore, the fluid solver can be seamlessly integrated into the LSTM neural architecture and trained as a whole differentiable program to achieve better learning performance.

### Contact model

Except the flow loading, VFs are also subjected to the collision forces between each other during the phase of vocal closure. In current study, left-right symmetry of VFs vibration is assumed and only the left side of VF was modeled. A penalty force contact model is applied at the midline. A contact pressure along the lateral direction is computed using Eq. (7):^46^

7 $${p}_{c}={k}_{c1}{dx}\left(1+{k}_{c2}{{dx}}^{2}\right).$$

where ${k}_{c1}$ and ${k}_{c2}$ are the contact coefficients, and ${dx}$ represents the penetration distance crossed the midline.

### Discrete PINN architecture

The overall PINN algorithm is illustrated in Supplementary Fig. 1. A detailed network layout is illustrated in Supplementary Fig. 2. The inputs of the network are the sequential 2D profiles in time extracted from the high-speed images. The network consists of a Long short-term memory (LSTM)-based recurrent encoder-decoder connected with a fully connected neural network (FCNN). Specifically, the LSTM encoder first encodes the whole 2D image sequence into a hidden vector, which is then passed to the LSTM decoder as the initial hidden state and cell state. Moreover, the decoding LSTM also takes the 2D profile at each time step as the input features, and it will output a sequence of hidden vectors to the following FCNN, which predicts the time history of modal coefficients ${b}_{j}\left(t\right)$ of the structure eigenmodes. The network-predicted ${b}_{j}\left(t\right)$ are then used to reconstruct full 3D dimensional shapes using Eq. (8):

8 $$\left\{X\left(t\right)\right\}=\left\{{X}_{0}\right\}+\left\{U\left(t\right)\right\}.$$

where $\left\{{X}_{0}\right\}$ represents the initial shape of VF and $\{X\left(t\right)\}$ represents the current shape of VF. A fully differentiable projection operation will be applied on the reconstructed 3D shapes to obtain the 2D projected profiles. Thanks to the differentiable computer program, the data loss, ${L}_{d},$ is constructed as the difference between the predicted and measured 2D profiles and the gradients can be back-propagated to trainable network parameters. The reconstructed 3D shapes are also used to compute the glottal area $A(y)$ and penetration distance *dx*. Using Eqs. (5) and (7), both fluid pressure and contact pressure can be computed, which constitutes the overall fluid loading $\{F(t)\}$. Using the predicted ${b}_{j}\left(t\right)$ and $\{F(t)\}$, the equation loss ${L}_{e}$, can be computed by summing the residual of Eq. (4) over all the eigenmodes utilized. The total loss constitutes the data loss and equation loss using Eq. (9):

9 $${L}_{f}={W}_{e}{L}_{e}+{W}_{d}{L}_{d}.$$

where ${W}_{e}$ and ${W}_{d}$ stand for the weight of the equation and data loss, respectively. It is worth noting that, every intermediate subroutines of the whole process is differentiable, which enables the neural network training in the reduced eigenspace with indirect (partial) 2D observation data.

After the convergence, other physical quantities such as ${f}_{0}$, flow rate, mucosal wave-speed, glottal opening, SPL, acoustic power, medial surface pressure, contact surface area and shape, stress, etc. can be computed, which could provide new metrics for better diagnosis and deeper insight into the underlying mechanisms. The structure of the hybrid algorithm is shown in Supplementary Fig. 1.

### Network hyperparameters

For the cases presented in this study, we used 128 features in the hidden state with 1 hidden layer for LSTM. The FCNN is a standard four-layer multilayer perceptron (MLP) with residual connections and layer norms, and each layer has 128 neurons. The output layer of the MLP takes 128-dimensional hidden features and predicts ${N}_{i}$-dimensional eigen coefficients. The ReLU activation function is used for the entire network. For the training, Adam optimizer is adopted with the ReduceLROnPlateau scheduler to dynamically adapt the learning rate when trapping at local minima. The initial and minimum learning rate were set to be $1.0\times {10}^{-2}$ and $5.0\times {10}^{-5}$, respectively. The weight of equation and data loss are ${10}^{4}$ and ${10}^{-5}$, respectively. We choose the parameters to make the magnitudes of the data loss and equation loss to be comparable. These hyperparameters have not been specifically tuned, and further fine-tuning may further improve the learning performance.

### Dataset preparation

To demonstrate the ability of the presented algorithm, we examined the performance of the algorithm on two datasets: synthetic canine simulation and experimental pigeon data.

For the synthetic canine dataset, we simulated the flow-induced vibration of the left VF (Fig. 1b) using 1D Bernoulli flow solver coupled with 3D Navier equation, assuming the left-right symmetry of VF vibration to reduce the training computational cost. The VF geometry was discretized into 20643 4-node tetrahedral elements. The VF was modeled as a two-layered structure: a cover layer and a body layer, both of which were modeled using transversely isotropic materials. (The material properties are listed in Supplementary Table 1). Rayleigh damping are used with the parameters: α = 60.0 s and β = 6.0$\times$10^−5^ s^−1^. The 3D solid dynamics are solved using the finite element method. The glottal flow channel was discretized into 100 horizontal sections, where Bernoulli’s equation was solved. The subglottal pressure of 1.0 kPa was considered, and the air density was 1.1 kg/m^3^. The simulation was conducted for 200 ms.

For the experimental pigeon datasets, we used the biological data from a previous study^57^, in which high-quality kinematic data of the lateral vibratory masses (LVMs, the VFs analog in syrinx) in in vitro self-oscillating rock pigeon syringes was obtained. The detailed information of the experiment can be found in ref. ^57^. Briefly, the anatomical models were constructed using DiceCT scans on 4 syringes. Figure 4b shows a scan for subject 1. The vibrations of the LVMs were captured using high-speed videos from the frontal view and their 2D profiles were annotated in each frame. The annotation can be approximated by the intersection of the mid-coronal plane with the left LVM (The red line in Fig. 4c). The acoustics of sound production were measured simultaneously with the video recording. The key kinematics and acoustic quantities were reported. For all subjects, the subglottal pressure, air density, number of sections in the flow direction, and number of epochs for training are the same as those of the canine simulation. The network predicts the modal coefficients of the first 50 vibration modes to reconstruct the 3D shape. Other information for the training of synthetic (canine) and experimental (pigeon) cases are summarized in Supplementary Table 2.

### Acoustic analysis

To measure the acoustic pressure, we used the linear source-filter theory^58^ by assuming that the vibration has not been affected by the acoustic pressure and by considering a monopole source of sound:

10 $${p}^{{\prime} }=\frac{{\rho }_{{air}}}{4\pi r}\frac{{dQ}}{{dt}}.$$

where ${p}^{{\prime} }$ is the acoustic pressure, $r$ stands for the distance from the source of the sound, and $\frac{{dQ}}{{dt}}$ represents the first temporal derivative of the flow rate.

To compare the acoustic features of the model with the experimental data^57^, we followed the same steps and calculated SPL and acoustic power from the acoustic pressure. The acoustic pressure was resampled to 48 kHz, and then low-pass filtered at 20 kHz. SPL, which is commonly used to indicate the strength of acoustic wave, was defined at 1 meter from the source as follows:

11 $${SPL}=20\log_{10} \left(\frac{p}{{p}_{{ref}}}\right)+{TL}.$$

where ${p}_{{ref}}$ = 2.0$\times$10^−5^ Pa, $p$ is the root-mean-square of the pressure, and ${TL}$ stands for transmission loss calculated by ${TL}=20\log _{10} \left(d\right)$ with $d$ = 12 cm. Moreover, acoustic power was calculated by

12 $${P}_{A}={AI}.$$

where $I$ is the sound intensity, and $A$ is the area of sound radiation ($A=4\pi {d}^{2}$).

### Statistics and reproducibility

In Fig. 3b, c, the standard deviation was calculated using 16 data points within one vibration cycle. In Fig. 5b, the standard deviation was calculated using data derived from 4 independent pigeon subjects. When comparing the prediction of the network model with the ground truth, the training was not repeated for statistical analysis because the results are highly reproducible. Repeated runs of the training are expected to produce statistically the same results, given convergence is achieved. When applying the neutral network model to a different tissue model, the 3D error of the prediction is expected to be comparable to what is presented in this paper for the synthetic data. For experimental datasets, the accuracy also depends on how well the 2D profiles are segmented from imaging data.

### Reporting summary

Further information on research design is available in the Nature Portfolio Reporting Summary linked to this article.

## Supplementary information

Supplementary Information

Description of Additional Supplementary Files

Supplementary Data 1

Supplementary Data 2

Supplementary Data 3

Supplementary Data 4

Supplementary Data 5

Supplementary Data 6

Reporting Summary

## Data Availability

The major output datasets generated and analyzed during the current study are available as Source Data File in Supplementary Data 1–6.

## Code availability

The custom code for this study is available at https://github.com/xudongzheng77/pinn2022^59^.
