# Supplementary material for: Predicting 3D soft tissue dynamics from 2D imaging using physics informed neural networks
Source: Commun Biol. 2023 May 18;6:541. doi: 10.1038/s42003-023-04914-y (PMC10199019; doi:10.1038/s42003-023-04914-y)
Supplement: Supplementary file 1 — Supplementary Information [file 42003_2023_4914_MOESM1_ESM.pdf]

Supplementary Information

Supplementary Table 1. Material property of the canine VF tissues for synthetic data generation

	E [kPa]	E' [kPa]	G [kPa]	μ [-]	μ' [-]
Body	4.76	95.24	23.81	0.9	0.0
Cover	1.33	26.70	6.68	0.9	0.0

Supplementary Table 2. Details of training parameters of synthetic (canine) and experimental (pigeon) cases

Case	Number of 2D profiles	α [s]	β [1/s]	k_{c1} [Pa/cm]	k_{c2} [cm^{-2}]
Canine	20	60.0	6.0×10^{-5}	(-)	(-)
Subject 1	38	75.4	1.3×10^{-4}	2.0×10^{4}	2.0×10^{5}
Subject 2	24	75.4	1.3×10^{-4}	1.0×10^{2}	1.0×10^{3}
Subject 3	38	26.4	3.4×10^{-5}	2.0×10^{4}	2.0×10^{5}
Subject 4	45	77.9	8.2×10^{-5}	1.0×10^{4}	1.0×10^{5}

Supplementary Figure 1. Structure of the discrete PINN-differentiable programming algorithm.

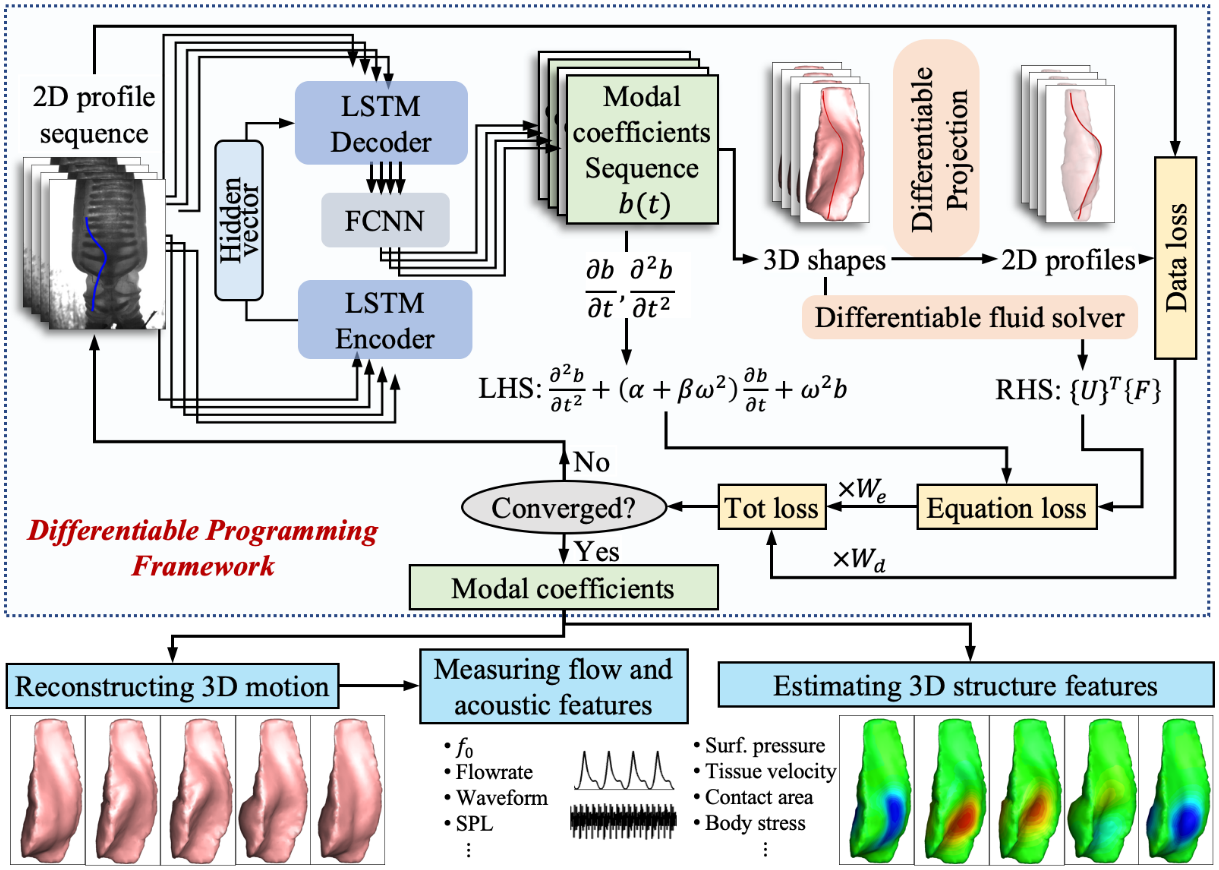

The input of the neural network is sequential 2D profiles in time. The neural network is built based on the Encoder-Decoder LSTM connected to the FCNN. The 3D shape is reconstructed from the predicted modal coefficients, which are projected to 2D observation space through differentiable projection operations to construct the data loss using 2D images. Equation loss is formulated by the force acting on the VF and the derivatives of the modal coefficients, based on a differentiable fluid solver. The combination of data and equation loss with proper weights forms the loss function. After the convergence, the final 3D motion is reconstructed, flow (e.g., f_0, flow rate, mucosal wave-speed, glottal opening, etc.) and acoustic (e.g., SPL, acoustic power, etc.) features are measured, and 3D features such as medial surface pressure, contact surface area and shape, stress, etc., which are difficult to be experimentally measured, are estimated.

Supplementary Figure 2. Expanded view of the whole network structure.

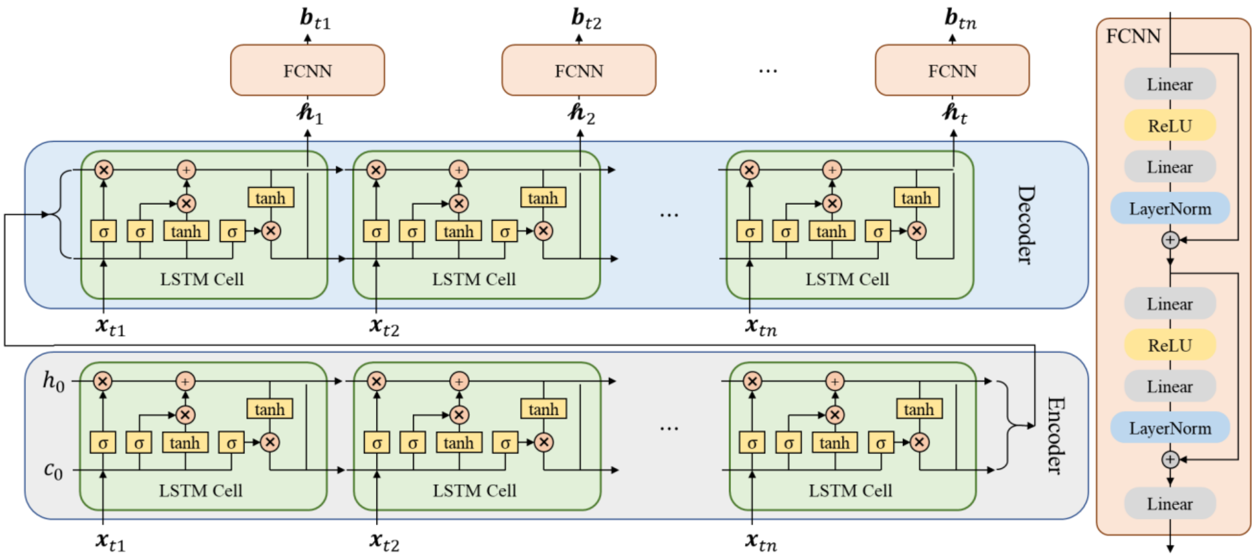

Note that each element in the input sequence $\boldsymbol{x}_t$ is a vector whose size corresponds to the number of points in the discretized 2D profile. And each element in the output sequence $\boldsymbol{b}_t$ is a vector whose size corresponds to the number of eigenmodes.
