# Supplementary material for: Predicting 3D soft tissue dynamics from 2D imaging using physics informed neural networks
Source: Commun Biol. 2023 May 18;6:541. doi: 10.1038/s42003-023-04914-y (PMC10199019; doi:10.1038/s42003-023-04914-y)
Supplement: Supplementary file 2 — Description of Additional Supplementary Files [file 42003_2023_4914_MOESM2_ESM.pdf]

Description of Additional Supplementary Files

File name: Supplementary Data 1

Description: The source data for Figure 1.

File name: Supplementary Data 2

Description: The source data for Figure 2.

File name: Supplementary Data 3

Description: The source data for Figure 3.

File name: Supplementary Data 4

Description: The source data for Figure 4.

File name: Supplementary Data 5

Description: The source data for Figure 5.

File name: Supplementary Data 6

Description: The source data for Figure 6.
